# Effects of the Anti-Tumor Agents Trabectedin and Lurbinectedin on Immune Cells of the Tumor Microenvironment

**DOI:** 10.3389/fonc.2022.851790

**Published:** 2022-03-01

**Authors:** Paola Allavena, Cristina Belgiovine, Elisabeth Digifico, Roberta Frapolli, Maurizio D’Incalci

**Affiliations:** ^1^ Department Immunology, IRCCS Humanitas Clinical and Research Center, Milan, Italy; ^2^ Department of Oncology, Istituto di Ricerche Farmacologiche Mario Negri IRCCS, Milan, Italy; ^3^ Department of Biomedical Sciences, Humanitas University, Milan, Italy

**Keywords:** tumor-associated macrophages, trabectedin, lurbinectedin, tumor micro-environment, immunity

## Abstract

Immune cells in the tumor micro-environment (TME) establish a complex relationship with cancer cells and may strongly influence disease progression and response to therapy. It is well established that myeloid cells infiltrating tumor tissues favor cancer progression. Tumor-Associated Macrophages (TAMs) are abundantly present at the TME and actively promote cancer cell proliferation and distant spreading, as well as contribute to an immune-suppressive milieu. Active research of the last decade has provided novel therapeutic approaches aimed at depleting TAMs and/or at reprogramming their functional activities. We reported some years ago that the registered anti-tumor agent trabectedin and its analogue lurbinectedin have numerous mechanisms of action that also involve direct effects on immune cells, opening up new interesting points of view. Trabectedin and lurbinectedin share the unique feature of being able to simultaneously kill cancer cells and to affect several features of the TME, most notably by inducing the rapid and selective apoptosis of monocytes and macrophages, and by inhibiting the transcription of several inflammatory mediators. Furthermore, depletion of TAMs alleviates the immunosuppressive milieu and rescues T cell functional activities, thus enhancing the anti-tumor response to immunotherapy with checkpoint inhibitors. In view of the growing interest in tumor-infiltrating immune cells, the availability of antineoplastic compounds showing immunomodulatory effects on innate and adaptive immunity deserves particular attention in the oncology field.

## Introduction

Trabectedin is a registered anti-tumor agent originally extracted from the marine organism *Ecteinascidia turbinate*, now synthetically produced by PharmaMar (Spain) (1). Trabectedin is used in the clinic for the second line treatment of soft tissue sarcoma (STS), especially liposarcoma and leiomyosarcoma, and for relapsed platinum-sensitive ovarian cancer, in combination with pegylated liposomal doxorubicin (2–5). Trabectedin was selected for its potent activity to kill cancer cells and efficiently block their proliferation by directly interacting with DNA. Its mechanism of action is complex and different from that of other anticancer agents: by binding to the minor grove, trabectedin directly interferes with activated transcription to poison the transcription-coupled nucleotide excision repair system and generates double-strand DNA breaks (6–14).

Further studies demonstrated that it mediates the displacement of oncogenic transcription factors from their target promoters, thereby affecting oncogenic signalling addiction (6, 13, 15, 16).

Besides its direct activity on cancer cells, a remarkable feature of trabectedin is its effects on the tumor micro-environment, in particular on cells of the mononuclear phagocyte system (monocytes/macrophages) as well as on the blood vessels. In this review we will focus on the peculiar tropism of trabectedin and of its analogue lurbinectedin on monocytes, macrophages and Tumor-Associated Macrophages (TAMs), and will discuss how these stromal-centered activities impact on their clinical anti-tumor efficacy.

## Cytotoxic Effect of Trabectedin on Mononuclear Phagocytes: *In Vitro* and *In Vivo* Studies

A distinguishing feature of trabectedin is its cytotoxic effect on mononuclear phagocytes. To distinguish the inhibitory activity of trabectedin on the cell cycle of proliferating cells from that on transcription factors, some years ago trabectedin was tested on non-proliferating immune cells. Circulating blood human monocytes were used as cells of choice, based on the fact that the transcription factor NF-Y, known to be inhibited by trabectedin (15) is expressed in monocytes and considered of major importance for their differentiation to mature macrophages (17, 18).

Quite surprisingly, monocytes exposed *in vitro* to nM concentrations of trabectedin proved to be highly affected and rapidly underwent apoptosis in a time frame of 24-48 hours. Other chemotherapeutic agents used in parallel as comparison (cisplatin and doxorubicin) had no such cytotoxic effect (19). Even more remarkably, this cytotoxic effect was highly selective for monocytes and macrophages, as neutrophils or T lymphocytes were not affected (19).

**Figure 1:**
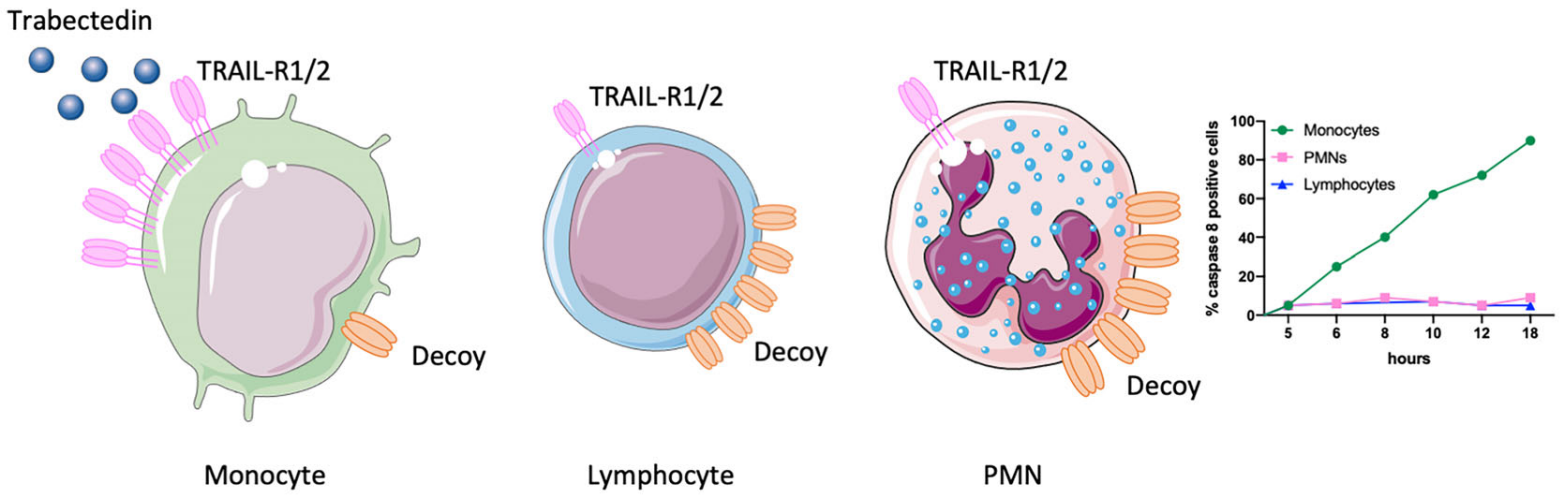
Selectivity of trabectedin for mononuclear phagocytes. TRAIL-R1 and R2 receptors are highly expressed in monocytes but not in T lymphocytes and neutrophils (PMNs) which indeed mainly express the non-signalling decoy receptor (TRAIL-R3). Thus, only monocytes are susceptible to trabectedin, which activates TRAIL-R1 and R2 and triggers a caspase 8-dependent apoptosis. This figure was made with Servier Medical Art templates, which are licensed under a Creative Commons Attribution 3.0. Unported License (https://smart.servier.com).

This finding stimulated a series of experiments to explain the selectivity of trabectedin for mononuclear phagocytes. It turned out that trabectedin rapidly triggers a caspase-dependent apoptosis where caspase-8 is activated within few hours (20). Caspase-8 is downstream of death membrane receptors, such as TRAIL receptors. The expression of TRAIL-R in the different leukocyte subsets was very informative to decipher the mechanism of trabectedin-induced apoptosis. TRAIL-R1 and R2 receptors were highly expressed in monocytes but not in neutrophils and T lymphocytes which, in turn, mainly expressed the non-signalling TRAIL-R3 (or decoy receptor) (21). Thus, the prevalent expression of functional TRAIL receptors in monocytes explained why only monocytes were susceptible to trabectedin, while neutrophils and T cells were spared by the decoy TRAIL-R3 that prevents activation of caspase-8 (**Figure 1**).

The analysis of TRAIL-R expression in leukocytes from human tissues revealed that in normal spleen and lungs, TRAIL-Rs were barely detectable, but in human tumor tissues from hepatic and mammary carcinoma, TRAIL-R2 was expressed in the majority of macrophages while it was absent in tumor infiltrating lymphocytes and neutrophils (21).

TRAIL-R molecules form a trimer with an internal space to lodge their ligand. Trabectedin is a small compound and likely is not directly binding to the trimer. However, it is well-known that some molecules, for instance the natural compounds Palmitate, Quercetin and some snail venoms are able to activate TRAIL-Rs and caspase-8 in a ligand-independent manner, through the upregulation and/or aggregation of death receptors (21–23).

Indeed, we found that *in vitro* treatment with trabectedin significantly upregulated the expression of TRAIL-R2 in monocytes and induced their aggregation into lipid rafts (21).

In view of these peculiar effects of trabectedin on mononuclear phagocytes it was of interest to demonstrate whether this compound was able to kill macrophages also *in vivo*, in particular Tumor-Associated Macrophages (TAMs) in experimental mouse tumor models. This issue bears particular importance because of the ambiguous liaison that TAMs have in the tumor tissue. In fact, it is now recognized that in established tumors myeloid cells of the innate immunity (especially macrophages) promote tumor progression and produce immunosuppressive factors that inhibit anti-tumor immune responses (24–28).

Using different pre-clinical tumor models, such as: fibrosarcoma, lung and ovarian cancer, the *in vivo* administration of trabectedin significantly and selectively reduced the number of blood monocytes, and that of macrophages in the tumor tissue (20). Interestingly, the only functional TRAIL-R expressed in mice (DR5) was selectively expressed on murine monocytes and TAMs and was virtually absent in neutrophils and lymphocytes. Therefore, the pattern of TRAIL-R expression in mice perfectly mimics that of human leukocytes (21).

Of interest, the percentage of splenic F4/80+ macrophages was also significantly decreased after treatment with trabectedin (20). Considering the population of MDSCs that expands in tumor-bearing animals, the monocytic component (M-MDSCs: GR1+ Ly6C^high^) was numerically reduced after treatment, while the granulocytic component (PMN-MDSCs: GR1+ Ly6C^low^) was not (20). This finding underlines - once more - the peculiar selectivity of trabectedin for the monocytic lineage. The phenotype analysis of MDSCs GR1+ in mouse tumor tissues revealed lower levels of DR5 compared to macrophages F4/80+ (21); however, others have reported that mouse and human MDSCs express functional TRAIL-Rs and are sensitive to TRAIL-mediated killing (29).

The ability of trabectedin to selectively kill macrophage *in vivo* in mice has been confirmed by several groups. In a mouse model of orthotopic pancreatic cancer, trabectedin strongly reduced the number of TAMs and of circulating monocytes, while neutrophils were not significantly affected (30). Other recent studies reported similar findings in mouse models of orthotopic osteosarcoma, melanoma and in skeletal metastasis from prostate cancer (31–33). In Ewing sarcoma, treatment with trabectedin alone had no efficacy on tumor growth, but the combination of trabectedin with oncolytic herpes virotherapy significantly improved mouse survival and this effect was related to a reduction in the number of TAMs and of Myeloid Derived Suppressor Cells (MDSC) (34). In hematological malignancies, trabectedin not only had cytotoxic effects on neoplastic cells but also induced the apoptotic death of associated myeloid cells (35–37). In another mouse model of acute promyelocytic leukemia, a recent study showed that depletion of bone marrow inflammatory monocytes with trabectedin prevented disease relapse (38).

The macrophage-depleting activity *in vivo* of trabectedin raised the question whether this effect was responsible, at least in part, for its *in vivo* anti-tumor efficacy. Using a fibrosarcoma variant that was resistant to the anti-proliferative activity of trabectedin, *in vivo* treatment with the drug resulted in a significant tumor growth inhibition, as the tumor-supporting TAMs were depleted by trabectedin. This effect was abolished by the adoptive transfer of fresh macrophages that promptly re-instated tumor growth post-treatment (20). These results strongly supported the conclusion that the anti-tumor activity of trabectedin relies both on its effects on cancer cells as well as on its cytotoxic activity on monocytes-macrophages.

Results in cancer patients are scarce, in spite of the fact that trabectedin is a registered compound. Patients with STS receiving trabectedin as single treatment have been studied for monocyte counts from circulating blood over therapy cycles; a decrease in monocytes indeed occurred in some patients within few days after each injection of trabectedin. Furthermore, in selected patients undergoing neo-adjuvant therapy with trabectedin, where tumor biopsies were also available, immunohistochemistry of tumor sections collected before and after therapy revealed a dramatic decrease of macrophage infiltration, reinforcing the finding that this compound is able to kill *in vivo* macrophages in tumor tissues (20).

## Trabectedin Inhibits the Production of Selected Inflammatory and Angiogenic Mediators: Impact on the Tumor Microenvironment

The study of trabectedin on immune cells held other surprises. It is well-known that its mechanism of action is not limited to binding of and damaging DNA, but also includes the transcriptional inhibition of selected genes. At low (non-cytotoxic concentrations) trabectedin inhibited the mRNA levels and production of specific inflammatory mediators in LPS-stimulated monocytes and macrophages such as IL-6 and several chemokines including: CCL2, CCL3, CCL7, CCL14 and CXCL8 (19, 39).

Importantly, other inflammatory cytokines such as TNF and IL-1 were not significantly inhibited, raising the question if this selectivity could be mechanistically ascribed to the ability of trabectedin to interfere with specific transcription factors. Inflammatory cytokines and chemokines are under the control of the master regulator NF-kB, but many mediators can be also activated by other transcription factors, such as activator protein (AP-1), SP-1 and Smad3 for CCL2 (40), and AP-1, cFOS, and CCAAT/NF-IL-6 for IL-6 (41).

Of these, AP-1 activates also TNF, that was unaffected by trabectedin. So far, the search for transcription factors common to CCL2 and IL-6 and not to TNF, and specifically affected by trabectedin has been unsuccessful.

Inhibition of inflammatory and angiogenic mediators was observed also in tumor cells. *In vitro* treatment with trabectedin decreased the production of CCL2, CXCL8, IL-6, VEGF and PTX3 by myxoid liposarcoma (MLS) primary tumor cultures and/or cell lines, and freshly isolated ovarian cancer cells from ascites (19, 39). In *in vivo* experiments, using a xenograft mouse model of human MLS, a marked reduction of human CCL2, CXCL8 and PTX3 after trabectedin administration was observed, demonstrating that this effect on tumor cells occurs also *in vivo* (39). A recent paper by Casagrande et al. reported that trabectedin inhibited the release of cytokines by tumor cells in Hodgkin lymphoma, including M-CSF, IL-6, IL-13, CCL5 and CCL17. Furthermore, treatment of mice bearing xenografts of Hodgkin lymphoma confirmed the *in vitro* findings, and residual tumors had fewer TAMs and a reduced vessel network (36).

It has been previously reported that trabectedin downmodulates the expression of ECM-related genes produced by TAMs and fibroblasts, such as collagen type 1, fibronectin, osteopontin and the matrix-metalloprotease-2 (MMP2) (42). These findings are of interest because they indicate that trabectedin may have an impact on the high matrix remodeling, a key feature of the cancerous stroma. Matrix degradation in tumor tissues, as well as in regenerating tissues, is known to release growth factors that are bound to ECM in an inactive forms. For instance, several angiogenic factors such as VEGF become activated during matrix remodeling and are available in the local environment. TAMs are an important source of pro-angiogenic factors in the TME; trabectedin significantly reduced the production of VEGF and angiopoietin-2 in macrophages and, accordingly, in tumor-bearing mice treated with the drug a clear decrease of the vessel network was observed (19, 20, 43). This TAM-mediated effect on angiogenesis was not the only impact of trabectedin on tumor angiogenesis: when mice were treated with the macrophage depleting agent liposomal-clodronate, there was no relevant impact on the vessel network, in spite of a significant inhibition of tumor growth (20). This finding indicated that trabectedin might have additional effects on blood vessels. Indeed, Taraboletti’s group demonstrated that trabectedin inhibited the matrix-invasion ability of endothelial cells and their morphogenetic branching (44). Mechanistically, trabectedin increased the expression of TIMP‐1 and TIMP‐2 that, by blocking the activity of the MMP enzymes, inhibited the proteolysis of ECM molecules, a required step in the process of matrix invasion (44). These anti-angiogenic effects of trabectedin were confirmed also in endothelial cells co-cultured with the conditioned medium of multiple myeloma cells, resulting in reduced capillary-like structures and fewer number of branching points (45). Other mechanisms of angiogenesis regulation acting *via* cancer cells were reported in mouse models of melanoma and myxoid liposarcoma, where trabectedin stimulated the tumoral expression of thrombospondin-1 (TSP-1), a major endogenous inhibitor of angiogenesis or of TIMP‐1 (31, 44). Overall, the anti-angiogenic activity of trabectedin occurs *via* different mechanisms, involving both a direct inhibitory effect on endothelial cells, as well as a reduction of the angiogenic potential of cancer cells and macrophages.

## The Analogue Lurbinectedin Shares With Trabectedin Similar Immunomodulatory Properties

Among several synthetized analogues of trabectedin, the compound lurbinectedin showed very promising anti-tumor activity *in vitro* and further on good efficacy in a broad range of clinical trials. Lurbinectedin has been approved by the Food and Drug Administration (FDA) in 2020 for the treatment of small cell lung carcinoma (46, 47).

Lurbinectedin contains the same pentacyclic skeleton of the tetrahydroisoquinoline rings, but it is structurally different as a tetrahydro beta-carboline replaces the additional tetrahydroisoquinoline of trabectedin. The structural similarity of lurbinectedin and trabectedin explains the similarity of the mode of action of the two drugs. Both trabectedin and lurbinectedin bind guanines at the N2 position, in the minor groove. They are both more cytotoxic against cells that are deficient in Homologous recombination (e.g., cells with mutations of BRCA genes) and less toxic against cells deficient in Nucleotide Excision Repair (10–12, 48, 49). Both drugs modify transcription regulation by displacing some oncogenic transcription factors from their target promoters (16, 50), and at high concentrations they cause degradation of RNA-polymerase II (51).

Lurbinectedin presents some interesting clinical features with pharmacokinetic and pharmacodynamic differences compared to trabectedin (51–53). Early phase I/II clinical studies demonstrated that, at equivalent administration schedules, the maximal-tolerated dose of lurbinectedin was more than 3 times higher than that of trabectedin, and the plasmatic Area Under the Curve (AUC) was 5-10 higher (51, 52). This difference emerged from the clinical investigations and was not anticipated based on preclinical data. In fact both *in vitro* studies on different cancer cell lines and *in vivo* studies in tumor bearing mice suggested a similar cytotoxic potency of the two drugs. The difference appears to be due to the different volume of distribution of lurbinectedin that in humans is four times lower than that of trabectedin (53, 54). The different volume of distribution is not only related to the different degree of lipophilicity of the two molecules, but also to a different binding affinity for alpha 1-acid glycoprotein (AGP). In fact equilibrium dialysis experiments showed that both compounds bind AGP, but the affinity of binding of lurbinectedin was much greater than that of trabectedin, KD values being approximately 8 and 87 nM for lurbinectedin and trabectedin respectively (55). The finding could be clinically relevant as AGP can be very variable in patients with cancer, particularly when tumors are at advanced stage and some inflammatory mechanisms are activated.

*In vivo* studies in preclinical mouse models confirmed that the anti-tumor efficacy of lurbinectedin was similar to that of trabectedin (11, 43); it therefore seemed plausible that the mechanisms of action of lurbinectedin also included macrophages of the tumor stroma as targets, in addition to cancer cells. The modulatory effects of lurbinectedin on immune cells was studied in parallel experiments with trabectedin. The results demonstrated that also lurbinectedin was able to significantly reduce monocyte viability at nM concentrations and to induce a caspase-dependent apoptotic cell death. Furthermore, similarly to trabectedin, this analogue inhibited selected inflammatory chemokines (CCL2 and CXCL8) and VEGF. In mouse tumor models, in addition to an excellent anti-tumor efficacy directed on cancer cells, lurbinectedin reduced the number of circulating monocytes, the tumor-infiltrating macrophages and the density of tumor vessels (43). To better analyze the comparison between trabectedin and lurbinectedin, a global gene expression analysis of drug-treated human monocytes was performed. Overall, the results indicated that the genes down- or up-modulated by trabectedin were also affected by lurbinectedin (43). As expected from previous results, several genes related to the inflammatory response, the DNA damage response and the apoptosis pathway were involved. Of interest several genes of the Rho GTPase family were significantly down-modulated by both compounds, a finding not appreciated in previous analyses (43).

RhoGTPases regulate intracellular actin dynamics and are involved in essential cell activities such as receptor signaling, cell adhesion, migration, and phagocytosis. Accordingly, monocytes treated with lurbinectedin or trabectedin showed a strongly impaired ability to migrate in response to chemo-attractants, such as the prototypical chemokine CCL2. This finding is important because the density of macrophages in tumors relies on the continuous migration of blood monocytes into tumor tissues (25, 27). Thus, not only trabectedin and lurbinectedin induce the apoptosis of monocytes/macrophages and inhibit their production of several biological mediators, the two drugs also have an impact on their mobilization and chemokine-induced attraction at tumor sites.

Overall, by comparing the two compounds for their activities on myeloid cells, and more in general on the TME, it can be concluded that lurbinectedin and trabectedin display very similar effects, *in vitro* and *in vivo*.

## Reprogramming the Immunosuppressive Myeloid Cells by Trabectedin: Potential for Combination With Immune Checkpoint Therapy

**Figure 2:**
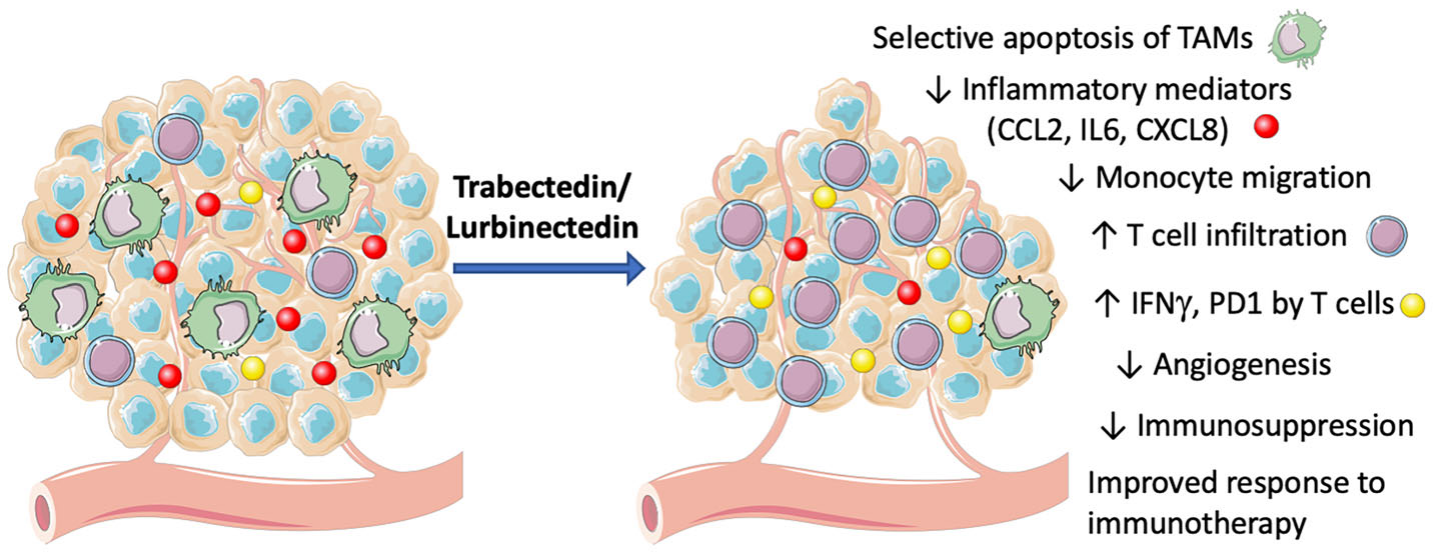
Mechanisms of action of trabectedin and lurbinectedin on the TME. Trabectedin and lurbinectedin share complex mechanisms of action on immune cells of the TME. They induce a selective apoptosis of TAMs, decrease monocyte migration and specific inflammatory mediators (CCL2, IL6, CXCL8). Moreover, trabectedin and lurbinectedin decrease angiogenesis and immunosuppression; they increase T cell infiltration and their expression of IFNγ and PD1, therefore improving the response to immunotherapy. This figure was made with Servier Medical Art templates, which are licensed under a Creative Commons Attribution 3.0. Unported License (https://smart.servier.com).

Depletion of macrophages by trabectedin and lurbinectedin may alleviate the TAM-mediated immune-suppression of adaptive anti-tumor responses in the TME, as depicted in **Figure 2**. This effect may have an important impact on the clinical response to immunotherapy. In fact, it is well known that immunosuppressive macrophages and related myeloid cells may impair the response to checkpoint inhibitors (25, 56, 57).

The potential effects of trabectedin on adaptive immune cells have been studied in preclinical models. Early findings already pointed out that in murine treated tumors the number of infiltrating T cells was increased (20). Recent studies specifically investigated the potential of trabectedin to modulate T lymphocytes in mouse cancer models. Analyses of tumor tissue in trabectedin-treated mice revealed a greater number of CD3+ and CD8+ lymphocytes by flow cytometry and immunohistochemistry (58). mRNA expression of several T cell-associated genes were significantly up-regulated after trabectedin, including the cytotoxic molecules granzyme B and perforin, the anti-tumor cytokine IFN γ and IFN-responsive genes such as MX1, CXCL10 and the checkpoint molecule PD-1 (58). These findings strongly indicate an activation of the T cell-mediated immune response upon macrophage targeting by trabectedin. Similar findings were reported in other studies. Borgoni et al. investigated the effects of trabectedin on tumor-infiltrating leukocytes in a genetic model of pancreatic cancer, a highly immunosuppressive tumor; treatment with trabectedin significantly reduced the immunosuppression in the TME: T lymphocytes sorted from treated tumors, showed an increased percentage of IFNγ+ Eomes+ and PD-1+ T cells, compared to untreated tumors, that were characterized by a higher proportion of IL10-expressing T cells. This switch towards an effector phenotype (IL10^low^/IFNγ^high^) indicated an important immunomodulatory outcome mediated by trabectedin on adaptive immunity and possibly leading to an anti-tumor phenotype (30). In a mouse model of osteosarcoma, trabectedin significantly reduced tumor burden and enhanced the number of infiltrating CD8+ T lymphocytes. Interestingly, also in this case T cells showed higher expression of the inhibitory molecule PD-1 (33).

Based on this finding, it was of interest to investigate whether the combination of trabectedin with anti-PD-1 checkpoint inhibitors improved the response to immunotherapy. Combination of trabectedin and anti-PD-1 showed increased efficacy in osteosarcoma and ovarian cancer mouse models (33, 59); using a mouse fibrosarcoma poorly responding to anti-PD-1 alone, an improved anti-tumor response was achieved when mice were pre-treated with trabectedin (58); in another study, depletion of myeloid cells combined with chemotherapy and PD-1 blockade, synergistically inhibited the progression of a murine leukemia (38).

In the fibrosarcoma model (58), an important aspect that emerged was the correct timing of the combination trabectedin and checkpoint immunotherapy. It was found that the best protocol was a sequential treatment (trabectedin first, followed by anti-PD-1), rather than a simultaneous administration. This sequential protocol will prepare a reprogrammed TME - with depletion of immunosuppressive macrophages - but above all will preserve the T cell activation induced by anti-checkpoint antibodies. In fact, T cell expansion can be blocked by the anti-proliferative action of trabectedin. Therefore, a reasoned timing of administration would consider using trabectedin first, and the immunotherapeutic treatment after some days.

These preclinical studies demonstrated that trabectedin positively remodulates the TME, likely through mitigation of the TAM-mediated immunosuppression, and facilitates T cell reactivation by anti-PD-1 antibodies. These findings have provided a rational to test the combination of trabectedin and anti-PD-1 antibodies in the clinic.

Indeed, some clinical trials of combination immunotherapy with trabectedin or lurbinectedin are ongoing (**Table 1**). In most cases patients are advanced and refractory to previous therapies. In phase 1/2 or phase 2 studies, patients with soft tissue sarcoma and ovarian cancer have been treated with trabectedin and anti-PD-1, anti-PD-L1 or anti-CTLA-4 (nivolumab, durvalumab, ipilimumab), while patients with small cell lung cancer (SCLC) have been treated with lurbinectedin and anti-PD-L1 or anti-CTLA-4 (atezolizumab, ipilimumab). In the study NCT03138161 (ClinicalTrials.gov) previously untreated sarcoma patients received a combination of trabectedin and ipilimumab or nivolumab, as a first line therapy. Some patients achieved good clinical responses without serious toxicity (60). (**Table 1**).

**Table 1:** Ongoing clinical studies using trabectedin or lurbinectedin in association with checkpoint blockade immunotherapy.

ClinicalTrials.gov Identifier	Clinical study	Combination therapy	Tumor type Published results
NCT03886311	Phase 2	trabectedin nivolumab talimogene laherparepvec*	Advanced sarcoma
NCT03138161	Phase 1 Expansion Phase 2	trabectedin nivolumab ipilimumab	Solid tumors (60)
NCT03085225	Phase 1b	trabectedin durvalumab	Advanced soft-tissue sarcoma Ovarian carcinoma (61)
NCT04253145	Phase 1/2	lurbinectedin atezolizumab	Small cell lung cancer
NCT04610658	Phase 1/2	lurbinectedin nivolumab ipilimumab	Small cell lung cancer

^*^Talimogene laherparepvec is an oncolytic herpes virus.

In the study NCT03085225, trabectedin was given in combination with durvalumab to advanced STS patients and to patients with ovarian cancer. Also in this study clinical responses were observed. Of interest, the tumor infiltration of CD8+ T cells was associated with prolonged survival in patients with ovarian carcinoma (61).

Overall, these promising results suggest the combination of trabectedin or lurbinectedin with checkpoint inhibitors deserves further assessment in the clinic.

## Conclusion

Trabectedin and lurbinectedin have multiple effects on immune cells of the tumor microenvironment and in particular on mononuclear phagocytes: at high concentrations they selectively induce a rapid caspase-dependent apoptosis in monocytes and TAM; at lower concentrations they inhibit the production of some inflammatory mediators with relevant activity for tumor biology; the two compounds also reduce monocyte adhesion and migration by inhibiting specific genes that organize the actin cytoskeleton. Furthermore, trabectedin and lurbinectedin hinder the production of angiogenic factors that are pivotal for tumor progression. Overall, in treated tumors there is a remarkable modulation of the TME with less immunosuppression and an increased presence of T lymphocytes. These conditions might be ideal to better respond to immunostimulatory approaches, such as checkpoint blockade immunotherapies. Therefore, trabectedin and lurbinectedin are interesting compounds in oncology, both for their intrinsic anti-tumor activity and for their remodulating effects on immunity.

## Author Contributions

Writing the manuscript: PA, CB, ED, RF, and MD’I. Creation of image: ED. Reading and proofreading: PA, CB, ED, RF, and MD’I. The review has been approved by all authors.

## Conflict of Interest

The authors declare that the research was conducted in the absence of any commercial or financial relationships that could be construed as a potential conflict of interest.

## Publisher’s Note

All claims expressed in this article are solely those of the authors and do not necessarily represent those of their affiliated organizations, or those of the publisher, the editors and the reviewers. Any product that may be evaluated in this article, or claim that may be made by its manufacturer, is not guaranteed or endorsed by the publisher.
